# Severe Acute Respiratory Syndrome (SARS) in Singapore: Clinical Features of Index Patient and Initial Contacts

**DOI:** 10.3201/eid0906.030264

**Published:** 2003-06

**Authors:** Li-Yang Hsu, Cheng-Chuan Lee, Justin A. Green, Brenda Ang, Nicholas I. Paton, Lawrence Lee, Jorge S. Villacian, Poh-Lian Lim, Arul Earnest, Yee-Sin Leo

**Affiliations:** * Tan Tock Seng Hospital, Tan Tock Seng, Singapore

**Keywords:** Severe acute respiratory syndrome, SARS, Singapore, coronavirus, dispatch

## Abstract

Severe acute respiratory syndrome (SARS) is an emerging viral infectious disease. One of the largest outbreaks of SARS to date began in Singapore in March 2003. We describe the clinical, laboratory, and radiologic features of the index patient and the patient’s initial contacts affected with probable SARS.

Severe acute respiratory syndrome (SARS), an atypical pneumonia characterized by high rate of transmission to healthcare workers (*1*), began in Guangdong Province, China, in November 2002. The largest SARS outbreak to date began in Singapore in mid-March 2003 and was traced to a traveler returning from Hong Kong.

According to the World Health Organization, a suspected case of SARS is defined as documented fever (temperature >38°C), lower respiratory tract symptoms, and contact with a person believed to have had SARS or history of travel to an area of documented transmission. A probable case is a suspected case with chest radiographic findings of pneumonia, acute respiratory distress syndrome (ARDS), or an unexplained respiratory illness resulting in death, with autopsy findings of ARDS without identifiable cause (*2*). We describe the clinical features of the index patient in Singapore and the patient’s initial group of contacts affected with probable SARS.

## The Index Case

The patient with the index case of SARS in Singapore was a previously healthy 23-year-old woman of Chinese ethnicity who had stayed on the 9th floor of a hotel during a vacation to Hong Kong, February 20–25, 2003. A physician from southern China who stayed on the same floor of the hotel during this period is believed to have been the source of infection for this index patient and the index patients of outbreaks in Vietnam and Canada.

**Figure 1:**
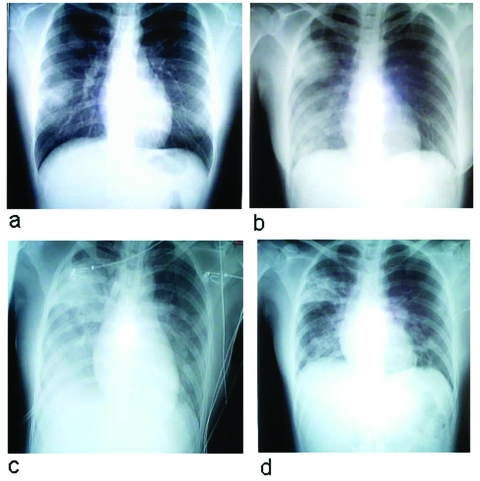
Chest radiographs of index patient with severe acute respiratory syndrome (SARS). a, day 5 of symptoms; b, day 10; c, day 13; d, day 15.

Fever and headache developed in the patient on February 25 and a dry cough on February 28. She was admitted to Tan Tock Seng Hospital, Singapore, on March 1. On admission she had oral temperature of 37.6°C and was lethargic. The chest was clear to auscultation. The remainder of her physical examination was normal. The total leukocyte count (2.7 × 10^9^/L), lymphocyte count (0.9 × 10^9^/L), and platelet count (102 × 10^9^/L) were reduced below normal laboratory ranges. Electrolytes and liver biochemistry results were normal. The chest x-ray showed patchy consolidation of both upper and lower lobes of her right lung (Figure1a). Blood cultures were sterile, and tests for urinary *Legionella* antigen, particle agglutination test for *Mycoplasma pneumoniae* antibodies, and complement fixation test for *Chlamydia* antibodies were negative. Immunofluorescence performed on nasopharyngeal aspirates for viral antigens of influenza virus A and B, parainfluenza virus, respiratory syncytial virus, and adenovirus was negative.

Intravenous levofloxacin, 500 mg once a day, was administered, but the patient’s temperature continued to spike up to 40°C, and the cough persisted. On day 5 of hospitalization, she became breathless and required supplemental oxygen. Sequential chest x-rays showed progressive, extensive involvement of the right lung, with new infiltrates appearing on the left (Figure 1c). Liver enzymes became elevated, with an ALT of 200 U/L (7–36 U/L) and AST of 208 U/L (15–33 U/L); serum lactate dehydrogenase (LDH) levels rose to 1518 U/L (200–500 U/L). Intravenous vancomycin (1 g twice a day) and oral oseltamivir (75 mg twice a day) were added to the regimen. Nine days after admission, the patient began to improve clinically, the laboratory abnormalities returned towards normal, and the chest x-ray abnormalities stabilized and resolved. The patient has remained well.

Electron microscopy of the nasopharyngeal aspirates swab taken on day 7 of hospitalization showed viral particles of <100 nm with widely spaced, club-shaped surface projections characteristic of coronaviruses.

### Clinical Features of Contact Cases

When the index patient was seen in early March, the clinical features and highly infectious nature of SARS were not known. For the first 6 days of hospitalization, the patient was in a general ward, without barrier infection control measures. One of eight physicians who attended her became infected, as did 9 of approximately 30 nursing staff. SARS also developed in 1 of 12 patients in adjacent beds during her hospitalization and 9 of approximately 30 family members and friends who visited her during this time. Nineteen of these 20 patients were admitted to our hospital for treatment and isolation (1 was treated outside Singapore), and we recorded prospectively the clinical features of their illnesses with a standardized data collection form. In addition to demographic data, this form elicited information on occupation, date(s) of exposure to suspected cases, travel history after February 20, dates of onset of various symptoms, results of blood tests, and chest radiographic findings.

**Figure 2:**
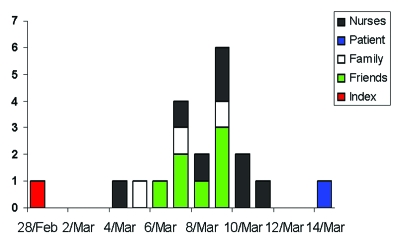
Index and contact cases of severe acute respiratory syndrome (SARS), by date of symptom onset.

The demographic profiles of the index and 19 contact cases are shown (Table 1). An epidemic curve of the index and contact cases is shown in Figure 2. Because most healthcare staff in our hospital are women, a high proportion of the case-patients (75%) were female. The median age of patients was 28 years. All were previously healthy, except one who had diabetes mellitus and end-stage renal failure and one who had a history of childhood asthma. One patient was a smoker. For seven patients who only had one exposure to the index patient, the median incubation period was 4 days (estimated range 2–8 days). For those with multiple exposures (13 patients), median incubation period was either 7 days (range 4–12 days, calculated from day 1 of exposure), or 5 days (range 3–9 days, calculated from midpoint of exposure period). The median period from onset of symptoms to admission was 6 days (range 0–9 days)

**Table 1:** Demographic description of patients with severe acute respiratory syndrome, Singapore

Demographics	No.
No. of men (%)^a^	5 (25)
No. of healthcare workers (%)^a^	9 (45)
Median age in years (range)	28 (19–73)
Median days from onset of symptoms to admission (range)	6.0 (0–9)

^a^N=20.

At admission, all patients had fever, sometimes accompanied by myalgia and headache (Table 2). Other symptoms, including dry cough, developed 2–4 days after onset of fever. Shortness of breath (when present) generally manifested in week 2 of illness. Apart from elevated temperature, results of physical examination were generally normal. The paucity of lung findings was often in striking contrast to the florid chest radiographic changes.

**Table 2:** Clinical features of severe acute respiratory syndrome

Symptom	No. (%) (N=20)
Fever	20 (100%)
Dry cough	15 (75%)
Myalgia	9 (45%)
Malaise	9 (45%)
Anorexia	9 (45%)
Shortness of breath	8 (40%)
Nausea/ vomiting	7 (35%)
Sore throat	5 (25%)
Diarrhea	5 (25%)
Headache	4 (20%)
Chills and rigors	3 (15%)
Rhinorrhea	3 (15%)

Laboratory investigations at admission are shown (Table 3). Lymphopenia, as defined by a cell count of <1.5 x 10^9^/L, was present in 18 of 20 patients. Other common laboratory abnormalities included leukopenia, thrombocytopenia, elevated LDH, mild hyponatremia, mild hypokalemia, and raised hepatic transaminases.

**Table 3:** Summary of severe acute respiratory syndrome signs and laboratory tests done at admission, Singapore, 2003

Test	Mean (SD)	Median (range)
Temperature (°C)	38.3 (0.9)	38.4 (37–40)
Oxygen saturation (%)	97.9 (1.8)	98 (92–100)
Leukocytes (4–10 x10^9^/L)	4.8 (2.2)	4.2 (2.5–10.6)
Lymphocytes (1.5–4.3 x10^9^/L)	0.9 (0.4)	0.7 (0.5–1.7)
Platelet count (160–390 x 10^9^/L)	159 (48.3)	151 (98–272)
Alanine aminotransferase (7–36 U/L)	40.6 (78)	17 (7–355)
Lactate dehydrogenase (200–500 U/L)	532.2 (260)	432 (306–1142)
Albumin (40–50 g/L)^a^	38.6 (5.1)	39 (25–46)
Globulin (25–40 g/L)	33.9 (4.6)	35 (25–45)
Urea (2–7.5 mmol/L)^a^	3.2 (1.5)	2.9 (1.9–8.5)
Creatinine (25–100 umol/L^)a^	65.4 (12.4)	65 (39–88)
Sodium (135–145 mmol/L)^a^	134.9 (2.8)	135 (131–141)
Potassium (3.5–5.0 mmol/L)^a^	3.5 (0.3)	3.6 (2.8–4)

^a^The laboratory results of Patient 20, who had end-stage renal failure, were not included in this table

**Figure 3:**
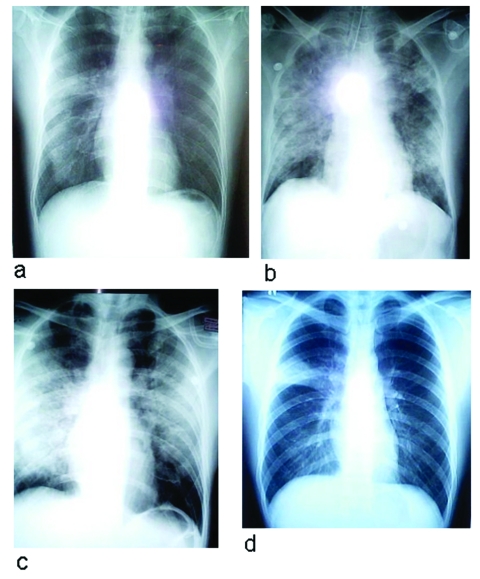
Chest radiographs of two patients with severe acute respiratory syndrome (SARS). a–c: radiographs of patient 5 showing progression of changes. a, day 8 of symptoms; b, day 13 of symptoms, d, day 14 of symptoms. He died on day 19 of this illness. d, chest radiograph, taken on day 8 of symptoms, of patient 12, with right upper lobe infiltrates resembling pulmonary tuberculosis (TB) but laryngeal swab cultures for TB were negative.

Abnormal chest radiographs were seen in 14 patients. Abnormalities developed in the remaining six patients by day 11 of illness. The most common pattern noted initially was interstitial infiltrates at the base of the right lung (11/20 patients). Right upper lobe infiltrates were seen in four patients, and involvement of both right upper and lower lobes was seen in three. One patient each had left lower lobe and bibasal infiltrates. Radiographic abnormalities rapidly progressed in all but one patient, and an ARDS-type picture developed in six patients, who subsequently required mechanical ventilation (Figures 1, 3).

Results of routine microbiologic cultures, serologic tests, and rapid antigen tests were universally negative. However, viral particles characteristic of coronavirus were found on electron microscopy examination of nasopharyngeal aspirates in 4 of 10 patients.

The clinical course of the illness is shown (Table 4). In 11 patients, oxygen saturation fell below 95%, and supplemental oxygen was instituted. Of these patients, six subsequently required mechanical ventilation for worsening respiratory failure. Clinical deterioration generally occurred in the week 2 of illness.

**Table 4:** Clinical course of severe acute respiratory syndrome (days after onset of symptoms) occurring up to April 10, 2003

Patient	Sex^a^	Age	Date of onset	Admission to hospital	First abnormal chest x-ray	Supplemental oxygen requirement	Mechanical ventilation	Fever resolved	Radiologic improvement	(Death)/ medically fit for discharge
1 (Index)	F	23	February 25	5	5	13	Not required	18	15	20
2	F	45	March 4	9	9	13	13	22	20	Hospitalized
3	F	46	March 5	6	6	11	13	Not seen	Not seen	(34)
4	M	25	March 6	9	9	Not required	Not required	12	14	19
5	M	50	March 7	8	8	8	10	Not seen	Not seen	(19)
6	F	27	March 7	5	8	Not required	Not required	16	16	21
7	F	23	March 7	5	5	Not required	Not required	10	13	15
8	M	40	March 7	6	6	8	10	Not seen	Not seen	(20)
9	F	28	March 8	7	9	Not required	Not required	11	12	13
10	F	26	March 8	7	7	11	Not required	14	14	17
11	M	27	March 9	8	8	Not required	Not required	12	14	17
12	M	39	March 9	7	11	Not required	Not required	13	15	18
13	F	19	March 9	7	7	13	Not required	20	17	23
14	F	73	March 9	7	7	10	11	12	18	20
15	F	23	March 9	6	6	11	Not required	16	16	19
16	F	35	March 9	0	9	Not required	Not required	11	12	13
17	F	22	March 10	7	11	Not required	Not required	13	14	17
18	F	30	March 10	5	9	Not required	Not required	11	14	15
19	F	22	March1 1	4	4	8	Not required	10	13	14
20	F	42	March 14	4	4	4	5	9	10	Hospitalized

^a^F, female; M, male.

All patients were initially treated with either levofloxacin or a combination of intravenous ceftriaxone and a macrolide once x-ray abnormalities were observed. Oseltamivir, 75 mg twice a day, and oral ribavirin, 20 mg/kg body weight three times a day, were prescribed to 6 and 14 patients, respectively. These antiviral drugs were started late in the course of illness: Most were prescribed from day 10 to day 14 of symptoms. Five patients in the intensive case unit were given corticosteroids (intravenous hydrocortisone, 100 mg every 6 hours, or intravenous methylprednisolone, 120 mg once a day).

Patients with ARDS were supported aggressively with intubation and mechanical, pressure-controlled ventilation with high positive end-expiratory pressures. Despite these and other supportive measures, three patients died of progressive respiratory failure.

Fever and laboratory and radiologic abnormalities resolved beginning from day 10 of illness. Patients with milder disease improved earlier. Of six patients who required mechanical ventilation, three died, and two improved sufficiently to be extubated. The last patient remains critically ill at the time of writing.

## Conclusions

Initial clinical features of SARS are nonspecific. Dry cough is common, although other symptoms of upper respiratory tract infection are unusual. Physical signs on chest examination are minimal, and chest radiograph may be normal on week 1 of illness. Laboratory tests often show lymphopenia, mild thrombocytopenia, and elevated liver enzymes. Therefore, in early stages, SARS may be hard to differentiate from other viral infections, and diagnostic delays may contribute to the spread of the epidemic. Early diagnosis relies on known history of potential exposure to SARS. Clinicians must maintain a high index of suspicion and be familiar with the rapidly changing epidemiology of this infection. Early diagnosis minimizes transmission.

In this initial cohort, radiologic changes eventually developed in all contacts with fever. However, the extent of chest x-ray changes and respiratory failure varied widely, and subsequent observations suggest a milder form of illness in which radiologic changes are not apparent. Whether asymptomatic infection can occur is unknown, but a more comprehensive description of the spectrum of clinical illness will only be possible when a diagnostic test is available. To this end, we are collecting serum from healthcare staff exposed to SARS patients.

The index patient infected at least 20 others over a period of several days. These cases were probably secondary rather than tertiary because the dates of symptom onset were very close and little interaction occurred between contacts. Patient 20 had a relatively late onset, but she was discharged from our hospital by March 6 and had no subsequent contact with other case-patients. Clearly, this infection is highly transmissible from person to person, and healthcare workers are particularly at risk (*3*–*5*). The precise routes of transmission in a healthcare setting need to be defined, although the predominance of right lower lobe findings on chest radiography suggests that droplet or airborne transmission is involved. However, were airborne transmission involved, we would have seen a much greater number of cases with weaker contact links to the index patient. We did not see any further transmission from this index patient after we implemented strict infection control measures involving use of N95 masks, gown, gloves, and handwashing before and after patient contact. Which components of this approach are responsible for the decrease in transmission is unclear.

A coronavirus was identified on electron microscopy of nasopharyngeal aspirates in our index patient and several of her contacts and is therefore likely to be responsible for this outbreak. This finding concurs with those in SARS outbreaks in other countries (*6*). Coronaviruses are widespread in the animal kingdom, and human coronaviruses are one of the main causes of the common cold. These viruses are also an important cause of pneumonia in military recruits (*7*). The aggressive nature of this disease suggests a new variant coronavirus; preliminary studies (*3*–*5*) confirm this hypothesis.

No effective treatment is known for this infection. We did not observe any obvious response to antibiotic therapy. However, until a specific diagnostic test is widely available, physicians should consider the use of empiric antibiotic therapy, including for atypical organisms in cases of probable SARS in which bacterial pneumonia cannot be definitely excluded.

Steroids were prescribed for five of six patients who had ARDS and were on mechanical ventilation. No perceived benefits accrued from this practice, although smaller doses were used than in Hong Kong (*4*,*5*).

A number of our patients received ribavirin, a broad-spectrum antiviral drug with known activity against some RNA viruses. However, we did not observe any obvious response to this drug, and several patients deteriorated in spite of its use. In contrast, a number of patients (including our index patient) recovered without use of ribavirin. Although some anecdotal reports of efficacy of ribavirin (when used in conjunction with steroids) exist (*4*,*5*), its efficacy is hard to judge for a condition that has potential for spontaneous recovery. Evidence from randomized controlled trials is needed before the use of ribavirin can be advocated for routine use in SARS patients.
